# Patient decision aid for trial of labor after cesarean (TOLAC) versus planned repeat cesarean delivery: a quasi-experimental pre-post study

**DOI:** 10.1186/s12884-021-04119-3

**Published:** 2021-09-23

**Authors:** Kartik K. Venkatesh, Suzanne Brodney, Michael J. Barry, Jamie Jackson, Kiira M. Lyons, Asha N. Talati, Thomas S. Ivester, Maria C. Munoz, John M. Thorp, Wanda K. Nicholson

**Affiliations:** 1 Division of Maternal Fetal Medicine, Department of Obstetrics and Gynecology, The Ohio State University, 395 W. 12th Ave., Floor 5, Columbus, OH USA; 2 Informed Medical Decisions Program, Massachusetts General Hospital, Boston, MA USA; 3 Division of General Obstetrics and Gynecology, University of North Carolina Chapel Hill, Chapel Hill, NC USA; 4 Division of Maternal Fetal Medicine, Department of Obstetrics and Gynecology, University of North Carolina Chapel Hill, Chapel Hill, NC USA

**Keywords:** Shared decision making, Decision aid, Trial of labor after cesarean, Cesarean delivery, Vaginal birth after cesarean, Telehealth

## Abstract

**Objective:**

To assess the impact of a web-based decision aid on patient-centered decision making outcomes among women considering a trial of labor after cesarean (TOLAC) versus planned repeat cesarean delivery.

**Methods:**

The Birth Decision Aid Study (B-READY) was a quasi-experimental pre-post study of two sequential cohorts. From June 18, 2018 to July 31, 2019, 50 women were enrolled in routine care, followed by 50 women who were enrolled in the decision aid group. Inclusion criteria were singleton pregnancies between 19/0 to 36/6 weeks, ≤2 prior cesareans, and no contraindications to TOLAC. The decision aid group viewed the online Healthwise® “Pregnancy: Birth Options After Cesarean” program. Both groups received the same birth options counseling and completed the same online assessment. Primary patient-centered outcomes were knowledge about birth options and shared decision making at online assessment, and informed, patient-centered decision making about her preferred mode of delivery at delivery admission.

**Results:**

Among 100 women participated in this study (50 per group), the mean gestational age at enrollment was 31 weeks, and 71% or 63/89 women who consented to delivery data abstraction had a cesarean delivery. Women in the patient decision aid group gained more knowledge (defined as score ≥ 75%) about birth options compared to those in the routine care group (72% vs. 32%; adjusted odds ratio, AOR: 6.15 [95% CI: 2.34 to 16.14]), and were more likely to make an informed, patient-centered decision (60% vs. 26%; AOR: 3.30 [95% CI: 1.20 to 9.04]. Women in both groups reported similar involvement in shared decision making, as well as satisfaction and values. More than 90% of decision aid users reported it was a useful tool and would recommend it to other TOLAC-eligible women.

**Conclusions:**

A web-based birth options patient-centered decision aid for TOLAC eligible women can be integrated into prenatal Telehealth and may improve the quality of decision making about mode of delivery.

**Trial registration:**

The study was registered with ClinincalTrials.gov and the ID# was NCT04053413. Registered 12 August 2019 – Retrospectively registered.

**Supplementary Information:**

The online version contains supplementary material available at 10.1186/s12884-021-04119-3.

## Background

Cesarean delivery is the most common surgery in the U.S. affecting nearly 1 in 3 pregnant women (1). Of the nearly 3.5 million women who deliver each year, 520,000 or 15% are faced with the decision whether to attempt a trial of labor after cesarean (TOLAC) or proceed with a planned repeat cesarean delivery [1]. Planned repeat cesarean delivery is a significant contributor to the cesarean delivery rate [2–5]. Over the last 20 years, fewer women are choosing to TOLAC, and rates have declined from 28% in 1996 to 12% in 2015 [6]. If all TOLAC eligible women had a vaginal birth after cesarean (VBAC), the cesarean rate in this population could drop from 70 to 25% [7].

Shared decision making (SDM) is a process of communication by which patients are informed and involved in decisions on their health care and is a key component of patient-centered care [8, 9]. The positive impact of shared decision making in obstetrics and gynecology has been demonstrated [10, 11], most recently in opioid prescribing after cesarean delivery and gynecologic procedures for benign disease [12, 13]. The American College of Obstetricians and Gynecologists (ACOG) emphasizes that decisions about the mode of delivery “should be made by the patient and her physician” and highlights the importance of eliciting patient preferences when discussing birth options [14]. TOLAC-eligible women want to discuss the risks and benefits of their birth options and be involved in the decision [15, 16]. However, current clinical practice is often of variable quality [17], improvised, and provider-driven [13, 18].

Patient decision aids are evidence-based tools that facilitate shared decision making, help make deliberate choices between healthcare options, provide accurate and unbiased information, and assist in clarifying values and treatment preferences [19–21]. A web-based patient decision aid using a shared decision making framework for TOLAC eligible women could optimize patient experience and possibly clinical outcomes [22]. Telehealth can serve as an efficient, cost-effective, sustainable, and scalable framework for the delivery of a patient decision aid [23].

Prior studies have not formally assessed a shared decision making framework, are now over a decade old, and primarily assessed impact on mode of delivery rather than patient-centered outcomes [24–26]. Recently, Kuppermann et al. published a multicenter RCT across of an electronic patient-centered decision aid among > 1400 TOLAC-eligible women, and found that the tool did not change the TOLAC rate and did not impact decision quality compared to routine care [27, 28]. There remains an unmet need for evidence-based shared decision support that utilizes Telehealth, which is rapidly increasing as a means of healthcare delivery [16, 20, 29, 30].

**Fig. 1:**
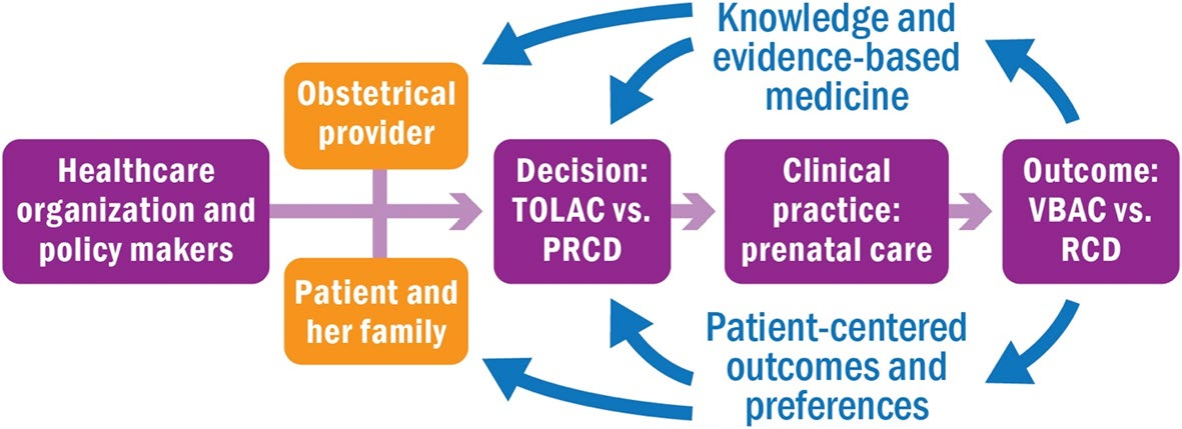
Conceptual diagram of shared decision making for TOLAC counseling. Footnote: This figure is adapted from a shared decision making conceptual framework for medical decision making as outlined by Sepucha et al. 2009 [31]. Abbreviations: TOLAC = trial of labor after cesarean, VBAC = vaginal birth after cesarean, PRCD = planned repeat cesarean delivery, and RCD = repeat cesarean delivery

Our objective was to assess the impact of a web-based decision aid as measured by patient-centered decision making outcomes for a TOLAC versus planned repeat cesarean delivery. We hypothesized that the patient decision aid would increase knowledge as well as shared and informed, patient-centered decision making, compared to routine care (Fig. 1) [31].

## Methods

### Study setting

Our Birth Decision Aid Study (also known as B-READY) was a quasi-experimental pre-post study with two sequential cohorts with online enrollment from June 18, 2018 to July 31, 2019 at prenatal offices affiliated with a tertiary care medical center. A quasi-experimental study involves the non-random assignment of participants into sequential pretest-posttest groups [32]. The study was approved by the Institutional Review Board at the University of North Carolina Chapel Hill. This study was funded by the University of North Carolina-Healthwise**®** Partnership Project on Birth Options Decision Aid, and the funder was not involved in conducting the study and writing the paper. Because the evidence base for patient decision aid effectiveness relies on accurate, complete, and high-quality reports of evaluation studies, we utilized the Standards for Universal reporting of patient Decision Aid Evaluation studies (SUNDAE Checklist) when reporting the findings from this study [21]. The study was registered with ClinincalTrials.gov (NCT04053413), retrospectively registered on 12/08/2019.

### Participants

We sequentially enrolled online 50 women in the routine care group followed by 50 women in the decision aid group from prenatal care practices in general obstetrics, maternal-fetal medicine, and midwifery affiliated with the University of North Carolina Women’s Hospital in Chapel Hill, NC. The a priori decision to enroll 50 women per group was consistent with the current recommended sample size justification for feasibility trials [33]. Study inclusion criteria were: 1) singleton pregnancy between 19/0 to 36/6 weeks’ gestation, 2) no more than two prior cesarean deliveries, and 3) no contraindications to TOLAC per current ACOG guidelines [14].

Potential participants were initially screened for eligibility using electronic health records (EHR). Eligible women were invited to participate via an email invitation and completed online screening questions to confirm eligibility. Up to three automated e-mail reminders were sent to initial non-responders after which women were categorized as unable to contact. Women were not required to deliver within our health network. For women who consented to delivery data abstraction, their intended route of delivery documented on delivery admission as well as their actual mode of delivery (cesarean or vaginal delivery) were later abstracted from the EHR by members of the study team (KKV, JJ).

### Online assessment and patient counseling

**Fig. 2:**
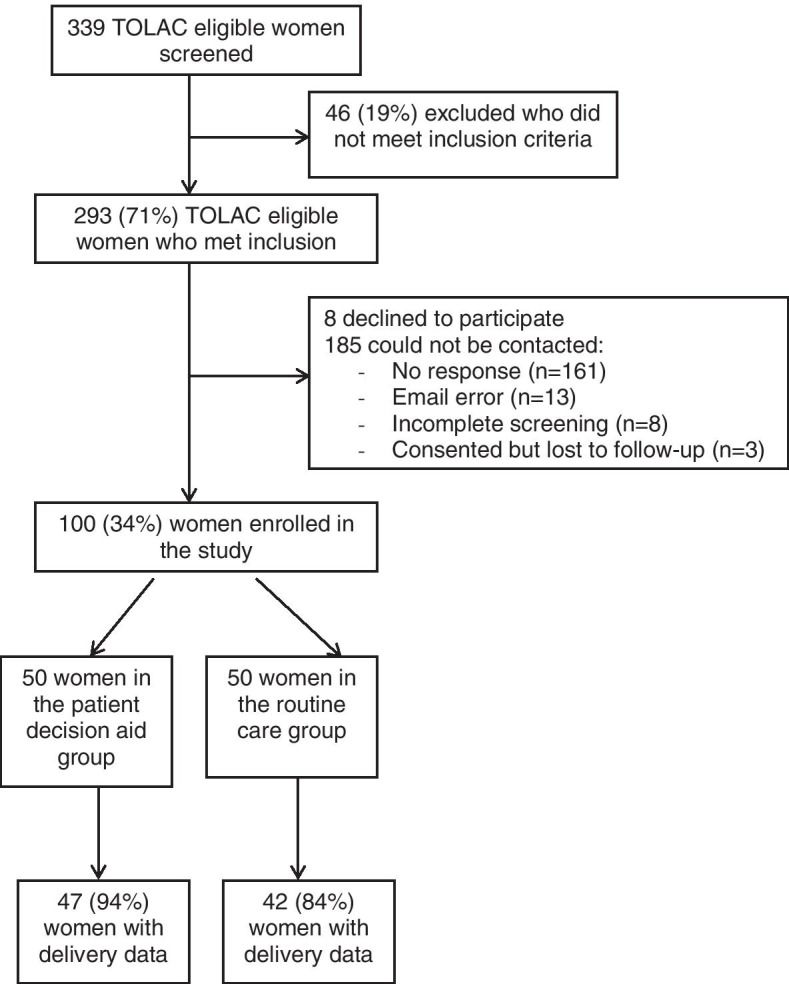
Flowchart of study participants

After completing the written informed consent form online, enrolled women completed a baseline online survey on their prior birth experiences, values, and preferences. After completing the baseline online survey, women in the routine care group received counseling on birth options as part of their regular prenatal care visit with their obstetric clinician and consequently completed a follow-up online survey assessment on their knowledge, values, preferences, and shared decision making experience (Fig. 2). Women in the patient decision aid group completed the same baseline online survey and viewed the web-based patient decision aid. At their subsequent prenatal visit, they received similar counseling on their birth options by their obstetric clinician as the routine care group. After the visit, they completed the follow-up online survey, which also included questions about the usefulness of the decision aid. Both groups received $10 following completion of the survey.

Prenatal counseling on birth options for both groups was only provided by obstetricians who perform cesarean deliveries. It is important to note that the shared decision making framework was not embedded into the clinical practice protocol for birth options counseling. Clinicians were not blinded to study participation, and women were encouraged to discuss their participation in the study with their clinician. Our institution had general guidelines for TOLAC counseling that all obstetric clinicians were encouraged to utilize and was available within the health system’s prenatal protocols which included the National Institute of Child Health and Human Development (NICHD) VBAC prediction tool [34]. No additional education, web tools, or decision support was given to the clinician. There were no institutional policies that restricted a TOLAC based on a patient’s predicted VBAC score. Use of the above resources was at the discretion of the clinician.

### Patient decision aid

The patient decision aid “Pregnancy: Your Birth Options After Cesarean” was available through a licensing agreement with Healthwise**®**, a 501(C)3 nonprofit organization (www.healthwise.org). Both patients and the public were stakeholders involved in its development, which included clinicians (obstetricians, family physicians, midwives, and nurses), women’s health advocates at the Childbirth Connection (www.childbirthconnection.org), and both pregnant women and women of childbearing age. The updated version dated March 16, 2017 was used in the current study. The patient decision aid was developed following the International Patient Decision Aid Standards Collaboration checklist (http://ipdas.ohri.ca/ipdas_checklist.pdf); and was certified by the Washington State Health Care Authority, which has been the primary patient decision aid-certifying organization in the United States (www.hca.wa.gov/about-hca/healthier-washington/patient-decision-aids-pdas#what-pdas-has-hca-certified). Neither Healthwise**®** nor any member of the content development team had relevant conflicts-of-interest.

The web-based patient decision aid was accessed via a computer or mobile device. The patient decision aid described key facts a TOLAC eligible woman should know. The benefits, harms, and consequences of both TOLAC and planned repeat cesarean delivery were presented, with icon arrays to communicate key numerical probabilities visually, such as the estimate for a successful VBAC. Personal stories of TOLAC eligible women were presented via video with available text transcript. The patient decision aid was tested with a focus group with consequent cognitive testing and health literacy assessment by the Rapid Estimate of Adult Literacy in Medicine—Short Form (REALM-SF) with a racially diverse sample of pregnant women, including those with public insurance [35]. The reading level of the patient decision aid calculated using the numerical formula from the Flesch-Kincaid tools was at the 6th grade.

### Study outcomes

Primary patient-centered outcomes included: 1) knowledge about birth options and 2) shared decision making at online survey assessment, and 3) informed, patient-centered decision making about preferred mode of delivery at delivery admission [31]. Patient satisfaction and values about birth options were assessed secondarily.

Patient knowledge about birth options was defined as “well informed” with a cutoff score of ≥75%, as previously described [36]. The 4-question knowledge test asked multiple-choice questions about key facts about TOLAC, including 1) post-operative recovery, 2) number of eligible women who attempt a TOLAC who have a cesarean delivery, and 3) risk and 4) clinical outcome of uterine rupture in labor (Additional file 1: Appendix Table A1).

The extent of shared decision making was assessed with the Shared Decision Making Process (SDMP) scale, which measured the extent the clinician informed and involved the patient about her birth options, as well as received input from the patient about what mattered to her (Additional file 1: Appendix Table A1). We summarized a decision process score that ranged from 0 to 4 by assigning 1 point each for 1) discussing the reasons for a test or treatment “a lot” or “some,” 2) talking about the reasons not to have a test or treatment “a lot” or “some,” 3) explaining that there was a choice to be made (yes), and 4) asking for the patient’s input (yes). This approach is consistent with prior studies by our group which used a similar SDMP scale [8, 37]. In line with the shared decision making model outlined by Charles et al. and Sepucha et al. [38, 39], we scored the answers so that the more complete the birth options discussion was of the pros and cons and the more women were informed about their choices and asked for their input, the decision was deemed to be more shared between the patient and her obstetric provider and more patient-centered was the decision making process [11].

To assess informed, patient-centered decision making on delivery admission, we calculated a summative score, as previously described by our group [36]. This assessment was performed retrospectively and participants were not assessed in real-time (i.e., at delivery admission). First, a knowledge score of ≥75% was used to define “well informed” (i.e., ≥3 correct of 4 questions). Second, a variable was generated by comparing patients’ preferred mode of delivery at online survey assessment (TOLAC, planned repeat cesarean delivery, not sure), a decision made weeks earlier, with their anticipated mode of delivery on admission. Patients who stated a preference for cesarean (i.e., planned repeat cesarean delivery) at survey assessment and received it on admission were considered to have “matched,” and those who stated a preference for a vaginal delivery (i.e., TOLAC) and received it were also considered to have matched. Patients who were “not sure” at either time point did not match. Patients who were both well informed and “matched” were counted as making an informed, patient-centered decision.

Secondarily, we assessed patient satisfaction and values in both groups. Satisfaction was evaluated based on the amount, clarity, and balance of information presented about birth options by the clinician. Values clarification was explicit, with the patient rating the importance of five key attributes of the decision (predictability, repeat surgery, pain management, return to activities, desire for vaginal birth) on a 0-to-10 or − 11 Likert scale.

Women in the patient decision aid group were asked about the number of video clips they viewed, usefulness of this aid when discussing birth options with their clinician, and whether they would recommend this online decision aid to other TOLAC eligible women. After study completion, patients received a summary of their ratings and delivery preferences, which they were encouraged to share with their clinician and family.

### Statistical analysis

We compared characteristics between women who enrolled in the current study and those who did not, and then between the patient decision aid and routine care groups using chi-square tests for categorical variables and student T-tests for continuous variables. Items with 4 response options (none, a little, some, and a lot) were consolidated into 2 (none or a little and some or a lot) for ease of presentation. Multivariable linear regression for continuous data (Shared Decision Making Process score) and logistic regression for dichotomous data (knowledge test score and informed, patient-centered decision score) were used to evaluate the association between study group and patient-centered outcomes. All models adjusted for prenatal care site (general OB/GYN, maternal-fetal medicine, and midwifery) and gestational age at study enrollment (continuous variable). Covariates were selected for adjustment based on a priori assessment and univariate statistical significance with a *p*-value < 0.10. All statistical analyses were performed using STATA (STATACORP, version MP 15.1, College Station, TX).

## Results

We initially identified 339 TOLAC eligible women from the EHR who met eligibility criteria and were sent email invitations (Fig. 2). Of those, 46/339 (19%) were excluded after online screening questions. Among the remaining 293 women, 100 agreed to participate and were enrolled in the study (50 in each group), and 193 women were eligible but did not enroll, 8/193 (4%) declined to participate and 185 (96%) did not respond to the email invitation. Women who did not enroll were significantly less likely to be of White race (50% vs. 78%; *p* < 0.001), have private insurance (54% vs. 83%; *p* < 0.001), and receive prenatal care with a maternal-fetal medicine specialist (46% vs. 33%; *p* < 0.01) compared to those who did enroll in the current study (Additional file 1: Appendix Table A2).

### Participant characteristics

The mean age was 33.6 (SD: 4.49) years, 78% had completed college or further education, and 22% were of non-white race (Table 1). The mean number of prior pregnancies was 2.7 (SD: 1.18), and 12% desired a future pregnancy. These characteristics did not differ between the two groups. However, the mean gestational age at the enrollment was 31 weeks (SD: 5.24), which was higher among women in the patient decision aid group versus routine care group (33 vs. 29 weeks; *p* < 0.001). Additionally, women in the patient decision aid group were more likely to receive prenatal care with maternal-fetal medicine (40% vs. 26%) and less likely to be with midwifery (14% vs. 34%) than the routine care group (overall *p* = 0.05).

**Table 1:** Participant characteristics overall and by study group at enrollment

Characteristic	Overall	Routine care	Patient decision aid	***P***-value^**1**^
	***N*** = 100	***N*** = 50	***N*** = 50	
	N (%)	N (%)	N (%)	
**Maternal age, mean (SD), years**	33.6 (4.49)	34.0 (4.66)	33.2 (4.32)	0.35
**Education, 4-year college or greater,*n*** **= 99**	78 (78.0)	41 (82.0)	37 (75.5)	0.43
**Self-reported race**				
White	78 (78.0)	39 (78.0)	39 (78.0)	0.87
Black	14 (14.0)	7 (14.0)	7 (14.0)	
Latina	5 (5.0)	3 (6.0)	2 (4.0)	
Other	3 (3.0)	1 (2.0)	2 (4.0)	
**Insurance status**				
Private	83 (83.0)	40 (80.0)	43 (86.0)	0.42
Government	17 (17.0)	10 (20.0)	7 (14.0)	
**Prenatal care site**				
General OB/GYN	43 (43.0)	20 (40.0)	23 (46.0)	0.05
Maternal-Fetal Medicine	33 (33.0)	13 (26.0)	20 (40.0)	
Midwifery	24 (24.0)	17 (34.0)	7 (14.0)	
**Gestational age, mean (SD), weeks**	31.2 (5.24)	29.1 (4.46)	33.2 (5.23)	< 0.001
**Number of prior pregnancies, mean (SD)**	2.76 (1.18)	2.84 (1.11)	2.68 (1.25)	0.50
**Prior pregnancy outcome,*n*** **= 99**				
Singleton	89 (89.9)	45 (90.0)	44 (89.8)	0.57
Twin or higher order gestation	9 (9.1)	5 (10.0)	4 (8.1)	
Stillbirth	1 (1.0)	0 (−-)	1 (2.0)	
**Desires a pregnancy in the future**	12 (12.0)	6 (12.0)	6 (12.0)	0.99

^1^*P*-value compares routine care vs. patient decision aid groups (chi-square for categorical variables and Student’s T-test for continuous variables)

### Patient-centered outcomes

 Women in the patient decision aid group demonstrated greater knowledge (test score ≥ 75%) about birth options compared to the routine care group (36/50, 72% vs. 16/50, 32%; adjusted odds ratio, AOR: 6.15 [95% CI: 2.34 to 16.14]) (Tables 2 and 3). Additionally, women in the patient decision aid group were more likely to make an informed, patient-centered decision about their preferred mode of delivery (28/47, 60% vs. 11/42, 26%; AOR: 3.30 [95% CI: 1.20 to 9.04]. Shared decision making was similar between both groups. Further adjustment for self-reported race, age, and education did not affect the above associations between the patient decision aid and the patient-centered outcomes.

**Table 2:** Frequency of shared decision making, patient knowledge, and making an informed, patient-centered decision comparing routine care vs. patient decision aid groups

Outcome measures	Routine care	Patient decision aid	***P***-value^**4**^
	***N*** = 50	***N*** = 50	
**Primary outcomes:**			
**Shared Decision Making Process (SDMP) score, median (IQR)**^b.c^**(*n*** **= 99)**	3 (2 to 4)	3 (2.5 to 4)	0.46
**Patient knowledge**^d^			
Mean score (SD)	49.5 (23.41)	78.5 (23.15)	< 0.001
Score ≥ 75%, n(%)	16 (32.0)	36 (72.0)	< 0.001
**Informed, patient-centered (IPC) decision on delivery admission**^a^	11/42 (26.1)	28/47 (59.6)	0.002
**Secondary outcomes:**			
**3Satisfaction**			
**Amount of information about birth options**			
Much less / a little less than wanted	15 (30.0)	11 (22.0)	0.36
About right/ more than wanted	35 (70.0)	39 (78.0)	
**Clarity of information about birth options**			
Poor / Fair	14 (28.0)	4 (8.0)	0.02
Good / Very Good	27 (54.0)	30 (60.0)	
Excellent	9 (18.0)	16 (32.0)	
**Balanced information about birth options**			
Strongly / slightly favors vaginal birth	15 (30.0)	14 (28.0)	0.07
Balanced	22 (44.0)	31 (62.0)	
Slightly / strongly favors repeat cesarean	13 (26.0)	5 (10.0)	
**Values**			
Know in advance the delivery date (1–11), mean (SD)	5.0 (3.87)	6.2 (3.44)	0.09
Avoid a cesarean delivery (1–11), mean (SD)	6.1 (3.66)	6.1 (3.89)	0.93
Little pain as possible during delivery (1–11), mean, (SD), n = 99	6.2 (3.12)	6.85 (2.64)	0.29
Return to usual activities quickly after birth (0–10), mean (SD)	7.9 (2.57)	7.7 (2.67)	0.65
Have a vaginal birth (0–10), mean (SD)	6.0 (3.56)	5.7 (3.56)	0.69

^a^*N* = 89 participants

^b^The Shared Decision Making Process Score assessed the clinician explaining birth options, including reasons for TOLAC and planned repeat cesarean delivery, and eliciting patient desires about mode of delivery

^c^Respondents received 1 point for each of the following responses: discussed pros = some or a lot, discussed cons = some or a lot, asked preference = yes, and choices explained = yes

^d^Knowledge assessed women’s knowledge of postoperative recovery, probability of repeat cesarean after TOLAC, and risk and implications of uterine rupture

^4^*P*-value compares routine care vs. patient decision aid groups (chi-square for categorical variables and Student’s T-test for continuous variables)

**Table 3:** Association between routine care versus patient decision aid groups and shared decision making, patient knowledge, and making an informed, patient-centered decision^a^

Study outcomes:	Unadjusted analysis	Adjusted analysis^b^
**Shared Decision Making Process (SDMP) score**		
β-coefficient, 95% CI^c^	0.15 (−0.25 to 0.56)	−0.02 (−0.45 to 0.41)
**Patient knowledge score ≥ 75%**		
Odds Ratio, 95% CI^c^	5.46 (2.31 to 12.87)	6.15 (2.34 to 16.14)
**Informed, patient-centered (IPC) decision**		
Odds Ratio, 95% CI^c^	4.15 (1.68 to 10.22)	3.30 (1.20 to 9.04)

^a^Final N for Shared Decision Making Process analysis = 99, patient knowledge = 100, and informed, patient-centered decision on delivery admission = 89

^b^Models adjusted for prenatal care site and gestational age at time of study enrollment

^c^The model for shared decision making process was developed with linear regression; and the models for patient knowledge and an informed, patient-centered decision were developed using logistic regression

With regards to satisfaction, women in the patient decision aid group were less likely to report poor clarity of information about birth options compared to the routine care group (8.0% vs. 28.0%; *p* = 0.02), but there were no differences between the two groups for amount of information and balanced information about birth options (Table 2). Patient values about birth options were similar between both groups.

### Delivery outcomes

Of the enrolled women, 89/100 consented to delivery EHR data abstraction (42/50 or 84% in the routine care group and 47/50 or 94% in the patient decision aid group). Delivery outcome data are presented for these 89 women, and 70% (63/89) delivered by repeat cesarean, which did not vary across both groups (70% for both). Overall, 17% (11/89) of women did not have the mode of delivery they had planned on delivery admission, and of these, all were planned TOLACs that resulted in repeat cesarean delivery (Table 4). Most (7/11, 63%) were for a labor dystocia (i.e., failed induction, failure to progress, or arrest of descent). Women in the patient decision aid group (12%, 4/47) were less likely to not have the mode of delivery they had planned on delivery admission compared to those in the routine care group (23%, 7/42).

**Table 4:** Clinical outcomes of women who planned to TOLAC on delivery admission and had a repeat cesarean delivery^a^

Participant	Study group	Informed patient-centered decision	Clinical indication for cesarean delivery	Complications
1	Patient decision aid	Yes	Patient decision	–
2	Patient decision aid	Yes	Concern for uterine rupture on admission	–
3	Patient decision aid	No	Failed induction	–
4	Patient decision aid	No	Non-reassuring fetal heart tracing	Spontaneous labor
5	Routine care	Yes	Failure to progress	Postpartum hemorrhage, uterine atony
6	Routine care	Yes	Failure to progress	–
7	Routine care	No	Arrest of descent	–
8	Routine care	No	Non-reassuring fetal heart tracing	–
9	Routine care	No	Failed induction	–
10	Routine care	No	Failed induction	Pre-eclampsia with severe features
11	Routine care	No	Failure to progress	Postpartum hemorrhage

^a^There were no women scheduled for a planned repeat cesarean delivery on delivery admission who had a consequent VBAC

Among women in the patient decision aid group, 12% (*n* = 6/50) reported not reviewing the online program. Among 88% (*n* = 44/50) of women who reviewed some or more of the patient decision aid, 95% reported they would recommend it to a TOLAC eligible friend, 97% reported that it was useful to understand their birth options, and 93% gave it an overall rating of good or better.

## Discussion

We found that a web-based patient decision aid on future birth options after cesarean can be integrated into prenatal Telehealth and may improve the quality of decision making about mode of delivery for TOLAC-eligible women. Those who received the patient decision aid were more likely to be well informed and receive their preferred mode of delivery on admission for delivery. Of concern is that only about a third of eligible women enrolled, and women who did were more likely to have completed college, be of White race, and have private insurance, which supports the need to better understand barriers to enrollment in a Telehealth program for pregnant women.

We found that women who received the web-based decision aid demonstrated greater knowledge of the risks and benefits and informed, patient-centered decision making about their birth options compared to those who received routine care. The patient decision aid did not increase shared decision making between patients and providers. Decision aids that integrate personalized prediction of TOLAC success with the elicitation of patient preferences and evidence-based information have been previously developed, but not widely implemented [20, 40–42]. Data from the last decade during an era of increasing Telehealth is notably lacking [24, 26], other than the mot recent multisite U.S. trial conducted by Kuppermann et al. among > 1400 TOLAC-eligible women [27]. The electronic patient centered intervention did not impact the primary outcome, which was the TOLAC rate, nor did it affect decision quality, which may have been due to the high baseline TOLAC rate (> 40%) and high level of reported decision quality in the study population [28]. In a randomized controlled trial in the United Kingdom conducted from 2004 to 2006 among 742 women, two computer-based programs on decisional conflict and mode of delivery (an informational program and a decision aid that combined patient preferences with predicted clinical outcomes versus routine care) reduced decisional conflict [25]. In a Canadian study conducted from 2005 to 2007, 131 TOLAC eligible women were assigned either to a decision aid or two evidence-based educational brochures, and found that both groups had less conflict around birth decisions post- compared to pre-intervention [26]. An Australian study conducted from 2001 to 2003 among 227 women found that a decision aid booklet describing risks and benefits of TOLAC versus planned repeat cesarean delivery improved knowledge and decreased decisional conflict [43]. In comparison to the current study, these prior decision aids, which are now nearly 15 years old, did not employ a shared decision making conceptual framework and were conducted prior to the current era of online Telehealth.

The TOLAC rate did not vary between groups, and it is important to note that the goal of the patient decision aid was to improve the *quality* of decision making through engagement in shared, informed decision making and valid counseling, and not necessarily to increase the uptake of vaginal delivery [31, 39]. While most women had a repeat cesarean delivery, those who made an informed, patient-centered decision on delivery admission were more likely to have a VBAC. Half the women in both groups planned to TOLAC at online survey assessment, and while this decreased by the time of delivery, the planned TOLAC rate was over three times the national average.

The characteristics of our study population (both patients and clinicians) could have affected the impact of the patient decision aid on the quality of decision making and anticipated mode of delivery. Studies that assess women’s preferences regarding mode of delivery after a prior cesarean have found that knowledge about the risks and benefits is associated with the likelihood to TOLAC [15, 16, 44]. Strength of preference for vaginal birth is predictive of the delivery mode ultimately undergone [45, 46]. In the current study, women on average had two or more prior deliveries, did not desire a future pregnancy, and had completed college. In addition to patient characteristics, hospital and clinician characteristics also affect mode of delivery preferences [47–50], including a dedicated antenatal clinic focused on counseling TOLAC-eligible women and more standardized intrapartum TOLAC management by a cadre of high-risk obstetricians [51], both of which existed at our institution. The current patient decision aid will need to be implemented and tested across regional settings with varying TOLAC rates and clinical practice models.

Strengths of this study include utilization of an online decision aid that can be integrated into the EHR as part of prenatal Telehealth. The patient decision aid was designed within a shared decision making framework. This study was conducted across a large healthcare system and different practice sites. Current approaches to TOLAC counseling can minimize patient involvement and be highly variable [22], leaving many women desiring more targeted information and structured guidance from their clinicians [17, 52]. This study begins to address growing need of patient decision aids using Telehealth [30].

A primary limitation is that the current study only included English-speaking women who were able to access a web-based patient decision aid. Most enrolled women had completed college, were of White race, and had private insurance, and those who did not enroll were more likely to be of non-White race and have public insurance, which suggests the need for further targeted outreach to these sub-populations of women [53]. The availability of Telehealth programs alone may be insufficient to overcome historic barriers to use and additional intervention may be needed to close the digital divide [54]. A second limitation is the difference in gestational age between study groups of almost 1 month. Prior research has highlighted the importance of timing of decision aids with regards to birth options counseling. To address these two limitations, we recommend that this small study be replicated in a randomized controlled trial of TOLAC-eligible women that ensures appropriate representation of women of diverse backgrounds.

There are several study limitations to note. This study did not provide any provider training in shared decision making, and did not elicit feedback from providers following birth options counseling. A systematic review of shared decision making interventions found that those programs that target both providers and patients were more efficacious than those that target just patients [19]. This was a pre-post study in which women were enrolled in sequential cohorts. Because group allocation was not randomized, there were some differences in baseline characteristics between the two groups as we describe above, which we addressed in multivariable analyses. This was a feasibility study in which enrollment was a priori set at 100 women [33]. We performed a post-hoc power calculation (80% power, one-sided test for a 20% increase in making a patient-centered decision among those in the decision aid group compared to the routine care group, with a baseline prevalence of 26% in the current routine care group), which would require a sample size of only 80 women. We recruited women across a wide gestational age range, and preferences about mode of delivery likely vary and evolve during the course of pregnancy. The current study was conduced at a single academic healthcare network, and institution-specific barriers may have affected utilization of the patient decision aid [10, 55]. However, women were recruited from across prenatal practice sites. More women in the current study desired a TOLAC compared to the national average, which may limit generalizability.

## Conclusions

We found that a web-based patient decision aid about birth options after a prior cesarean based on a shared decision making framework can be integrated into prenatal Telehealth and has the potential to increase the quality of decisions about mode of delivery. A larger randomized controlled trial is needed, which needs to reach sub-populations of women who may encounter barriers to accessing a Telehealth program. In an era of expanding Telehealth interventions [30], this study addresses the importance of evidence-based, patient-centered care based on shared decision making for birth options discussions among TOLAC eligible women.

## Supplementary Information

**Additional file 1.**

## Data Availability

The datasets generated and/or analyzed during the current study are not publicly available as additional analyses are ongoing. Requests for access to the original data can be obtained by contacting the corresponding author Dr. Kartik K Venkatesh by email at kartik.venkatesh@osumc.edu.
