# Supplementary material for: Patient decision aid for trial of labor after cesarean (TOLAC) versus planned repeat cesarean delivery: a quasi-experimental pre-post study
Source: BMC Pregnancy Childbirth. 2021 Sep 23;21:650. doi: 10.1186/s12884-021-04119-3 (PMC8461956; doi:10.1186/s12884-021-04119-3)
Supplement: Supplementary file 1 — Additional file 1. [file 12884_2021_4119_MOESM1_ESM.docx]

**APPENDIX**

| **Table A1. Participant shared decision making and knowledge score questions by study group** | | | |
| --- | --- | --- | --- |
|  | **Routine care** | **Patient decision aid** | **p-value** |
|  | **N=50** | **N=50** |  |
|  | **N (%)** | **N (%)** |  |
| **Shared Decision Making Process (SDMP) score questions (n=99)** |  |  |  |
| How much did you and the health care provider talk about the reasons you might want to plan a repeat c-section?  1 Not at all  2 A little  3 Some  4 A lot | 31 (62.0) | 33 (66.0) | 0.67 |
| How much did you and the health care provider talk about why you might not want to plan a repeat c-section?  1 Not at all  2 A little  3 Some  4 A lot | 35 (50.0) | 30 (60.0) | 0.31 |
| How much did you and the health care provider talk about the reasons you might want to plan a vaginal birth?  1 Not at all  2 A little  3 Some  4 A lot | 27 (54.0) | 29 (58.0) | 0.68 |
| How much did you and the health care provider talk about why you might not want to plan a vaginal birth?  1 Not at all  2 A little  3 Some  4 A lot | 25 (50.0) | 24 (48.0) | 0.84 |
| Did the health care provider explain that there were choices about how to plan your birth after having one or two previous c-sections?  1 Yes  2 No | 42 (85.7) | 46 (92.0) | 0.32 |
| Did the health care provider ask you what type of birth you wanted?  1 Yes  2 No | 45 (91.8) | 47 (94.0) | 0.67 |
| **Correct Answers (bolded) to Each Knowledge Score Question** |  |  |  |
| Which group of women is more likely to have trouble doing their usual activities in the first 2 months after they give birth? (offered answers below; only one correct in boldface)  1 **Women who have a C-section**  2 Women who have a vaginal birth  3 There is no difference  4 I am not sure | 44 (88.0) | 49 (98.0) | 0.05 |
| About how many women who try to have a vaginal birth after having a C-section will end up needing another C-section? (offered answers below; only one correct in boldface)  1 **Less than half**  2 About half  3 More than half  4 I am not sure | 17 (34.0) | 36 (72.0) | <0.001 |
| Think about women who had a C-section in the past and then try to have another baby vaginally. About how many of those women will have a scar from a past C-section break open during labor? (offered answers below; only one correct in boldface)  1 10 in 10,000  2 50 in 10,000  3 **100 in 10,000**  4 500 in 10,000  5 I am not sure | 6 (12.0) | 25 (50.0) | <0.001 |
| Think about women who had a C-section in the past and then try to have another baby vaginally. What happens if the woman is in labor and the scar from her past C-section breaks open? (offered answers below; only one correct in boldface)  1 She will still be able to give birth vaginally if she wants  2 **She will have to have an emergency C-section**  3 There is no way to predict what will happen  4 I am not sure | 32 (64.0) | 47 (94.0) | <0.001 |

| **Table A2. Characteristics of enrolled women vs. eligible women not enrolled** | | | |
| --- | --- | --- | --- |
| **Characteristic** | **Enrolled** | **Eligible and not enrolled** | **p-value^1^** |
|  | **N=100** | **N=193** |  |
|  | **N (%)** | **N (%)** |  |
| **Maternal age, mean (SD), years** | 33.6 (4.49) | 32.4 (5.12) | 0.05 |
| **Self-reported race**  White  Black  Latina  Other | 78 (78.0)  14 (14.0)  5 (5.0)  3 (3.0) | 96 (49.7)  60 (31.1)  0 (--)  37 (19.1) | <0.001 |
| **Insurance status**  Private  Government | 83 (83.0)  17 (17.0) | 103 (53.9)  88 (46.1) | <0.001 |
| **Prenatal care site**  General OB/GYN  Maternal-Fetal Medicine  Midwifery | 43 (43.0)  33 (33.0)  24 (24.0) | 84 (43.5)  88 (45.6)  21 (10.9) | <0.01 |
| ^1^p-value compares routine care vs. patient decision aid groups (chi-square for categorical variables and Student’s T-test for continuous variables). | | | |
